# Reporting Guidelines for Survey Research: An Analysis of Published Guidance and Reporting Practices

**DOI:** 10.1371/journal.pmed.1001069

**Published:** 2011-08-02

**Authors:** Carol Bennett, Sara Khangura, Jamie C. Brehaut, Ian D. Graham, David Moher, Beth K. Potter, Jeremy M. Grimshaw

**Affiliations:** 1 Ottawa Hospital Research Institute, Clinical Epidemiology Program, Ottawa, Canada; 2 Department of Epidemiology and Community Medicine, University of Ottawa, Ottawa, Canada; 3 Canadian Institutes of Health Research, Ottawa, Canada; 4 Department of Medicine, University of Ottawa, Ottawa, Canada; Medical Research Council, South Africa

## Abstract

**Background:**

Research needs to be reported transparently so readers can critically assess the strengths and weaknesses of the design, conduct, and analysis of studies. Reporting guidelines have been developed to inform reporting for a variety of study designs. The objective of this study was to identify whether there is a need to develop a reporting guideline for survey research.

**Methods and Findings:**

We conducted a three-part project: (1) a systematic review of the literature (including “Instructions to Authors” from the top five journals of 33 medical specialties and top 15 general and internal medicine journals) to identify guidance for reporting survey research; (2) a systematic review of evidence on the quality of reporting of surveys; and (3) a review of reporting of key quality criteria for survey research in 117 recently published reports of self-administered surveys. Fewer than 7% of medical journals (n = 165) provided guidance to authors on survey research despite a majority having published survey-based studies in recent years. We identified four published checklists for conducting or reporting survey research, none of which were validated. We identified eight previous reviews of survey reporting quality, which focused on issues of non-response and accessibility of questionnaires. Our own review of 117 published survey studies revealed that many items were poorly reported: few studies provided the survey or core questions (35%), reported the validity or reliability of the instrument (19%), defined the response rate (25%), discussed the representativeness of the sample (11%), or identified how missing data were handled (11%).

**Conclusions:**

There is limited guidance and no consensus regarding the optimal reporting of survey research. The majority of key reporting criteria are poorly reported in peer-reviewed survey research articles. Our findings highlight the need for clear and consistent reporting guidelines specific to survey research.

*Please see later in the article for the Editors' Summary*

## Abstract 2

Carol Bennett and colleagues review the evidence and find that there is limited guidance and no consensus on the optimal reporting of survey research.

## Editors' Summary

**Background:**

Surveys, or questionnaires, are an essential component of many types of research, including health, and usually gather information by asking a sample of people questions on a specific topic and then generalizing the results to a larger population. Surveys are especially important when addressing topics that are difficult to assess using other approaches and usually rely on self reporting, for example self-reported behaviors, such as eating habits, satisfaction, beliefs, knowledge, attitudes, opinions. However, the methods used in conducting survey research can significantly affect the reliability, validity, and generalizability of study results, and without clear reporting of the methods used in surveys, it is difficult or impossible to assess these characteristics and therefore to have confidence in the findings.

**Why Was This Study Done?:**

This uncertainty in other forms of research has given rise to Reporting Guidelines—evidence-based, validated tools that aim to improve the reporting quality of health research. The STROBE (STrengthening the Reporting of OBservational studies in Epidemiology) Statement includes cross-sectional studies, which often involve surveys. But not all surveys are epidemiological, and STROBE does not include methods' and results' reporting characteristics that are unique to surveys. Therefore, the researchers conducted this study to help determine whether there is a need for a reporting guideline for health survey research.

**What Did the Researchers Do and Find?:**

The researchers identified any previous relevant guidance for survey research, and any evidence on the quality of reporting of survey research, by: reviewing current guidance for reporting survey research in the “Instructions to Authors” of leading medical journals and in published literature; conducting a systematic review of evidence on the quality of reporting of surveys; identifying key quality criteria for the conduct of survey research; and finally, reviewing how these criteria are currently reported by conducting a review of recently published reports of self-administered surveys.

The researchers found that 154 of the 165 journals searched (93.3%) did not provide any guidance on survey reporting, even though the majority (81.8%) have published survey research. Only three of the 11 journals that provided some guidance gave more than one directive or statement. Five papers and one Internet site provided guidance on the reporting of survey research, but none used validated measures or explicit methods for development. The researchers identified eight papers that addressed the quality of reporting of some aspect of survey research: the reporting of response rates; the reporting of non-response analyses in survey research; and the degree to which authors make their survey instrument available to readers. In their review of 117 published survey studies, the researchers found that many items were poorly reported: few studies provided the survey or core questions (35%), reported the validity or reliability of the instrument (19%), discussed the representativeness of the sample (11%), or identified how missing data were handled (11%). Furthermore, (88 [75%]) did not include any information on consent procedures for research participants, and one-third (40 [34%]) of papers did not report whether the study had received research ethics board review.

**What Do These Findings Mean?:**

Overall, these results show that guidance is limited and consensus lacking about the optimal reporting of survey research, and they highlight the need for a well-developed reporting guideline specifically for survey research—possibly an extension of the guideline for observational studies in epidemiology (STROBE)—that will provide the structure to ensure more complete reporting and allow clearer review and interpretation of the results from surveys.

**Additional Information:**

Please access these web sites via the online version of this summary at http://dx.doi.org/10.1371/journal.pmed.1001069.

- More than 100 reporting guidelines covering a broad spectrum of research types are indexed on the EQUATOR Networks web site
- More information about STROBE is available on the STROBE Statement web site

## Introduction

Surveys are a research method by which information is typically gathered by asking a subset of people questions on a specific topic and generalising the results to a larger population [1],[2]. They are an essential component of many types of research including public opinion, politics, health, and others. Surveys are especially important when addressing topics that are difficult to assess using other approaches (e.g., in studies assessing constructs that require individual self-report about beliefs, knowledge, attitudes, opinions, or satisfaction). However, there is substantial literature to show that the methods used in conducting survey research can significantly affect the reliability, validity, and generalisability of study results [3],[4]. Without clear reporting of the methods used in surveys, it is difficult or impossible to assess these characteristics.

Reporting guidelines are evidence-based, validated tools that employ expert consensus to specify minimum criteria for authors to report their research such that readers can critically appraise and interpret study findings [5]–[7]. More than 100 reporting guidelines covering a broad spectrum of research types are indexed on the EQUATOR Network's website (www.equator-network.org). There is increasing evidence that reporting guidelines are achieving their aim of improving the quality of reporting of health research [8]–[11].

Given the growth in the number and range of reporting guidelines, the need for guidance on *how* to develop a guideline has been addressed [7]. A well-structured development process for reporting guidelines includes a review of the literature to determine whether a reporting guideline already exists (i.e., a needs assessment) [7]. The needs assessment should also include a search for evidence on the quality of reporting of published research in the domain of interest [7].

The series of studies reported here was conducted to help determine whether there is a need for survey research reporting guidelines. We sought to identify any previous relevant guidance for survey research, and any evidence on the quality of reporting of survey research. The objectives of our study were:

1. to identify current guidance for reporting survey research in the “Instructions to Authors” of leading medical journals and in published literature;
2. to conduct a systematic review of evidence on the quality of reporting of surveys; and
3. to identify key quality criteria for the conduct of survey research and to review how they are being reported through a review of recently published reports of self-administered surveys.

## Methods

### Part 1: Identification of Current Guidance for Survey Research

#### Identifying guidance in “Instructions to Authors” sections in peer reviewed journals

Using a strategy originally developed by Altman [12] to assess endorsement of CONSORT by top medical journals, we identified the top five journals from each of 33 medical specialties, and the top 15 journals from the general and internal medicine category, using Web of Science citation impact factors (list of journals available on request). The final sample consisted of 165 unique journals (15 appeared in more than one specialty).

We reviewed each journal's “Instructions to Authors” web pages as well as related PDF documents between January 12 and February 9, 2009. We used the “find” features of the Firefox web browser and Adobe Reader software to identify the following search terms: survey, questionnaire, response, response rate, respond, and non-responder. Web pages were hand searched for statements relevant to survey research. We also conducted an electronic search (MEDLINE 1950 – February Week 1, 2009; terms: survey, questionnaire) to identify whether the journals have published survey research.

Any relevant text was summarized by journal into categories: “No guidance” (survey related term found; however, no reporting guidance provided); “One directive” (survey related term(s) found that included one brief statement, directive or reference(s) relevant to reporting survey research); and “Guidance” (survey related term(s) including more than one statement, instruction and/or directive relevant to reporting survey research). Coding was carried out by one coder (SK) and verified by a second coder (CB).

#### Identifying published survey reporting guidelines

MEDLINE (1950 – April Week 1, 2011) and PsycINFO (1806 – April Week 1, 2011) electronic databases were searched via Ovid to identify relevant citations. The MEDLINE electronic search strategy (Text S1), developed by an information specialist, was modified as required for the PsycINFO database. For all papers meeting eligibility criteria, we hand-searched the reference lists and used the “Related Articles” feature in PubMed. Additionally, we reviewed relevant textbooks and web sites. Two reviewers (SK, CB) independently screened titles and abstracts of all unique citations to identify English language papers and resources that provided explicit guidance on the reporting of survey research. Full-text reports of all records passing the title/abstract screen were retrieved and independently reviewed by two members of the research team; there were no disagreements regarding study inclusion and all eligible records passing this stage of screening were included in this review. One researcher (CB) undertook a thematic analysis of identified guidance (e.g., sample selection, response rate, background, etc.), which was subsequently reviewed by all members of the research team. Data were summarized as frequencies.

### Part 2: Systematic Review of Published Studies on the Quality of Survey Reporting

The results of the above search strategy (Text S1) were also screened by the two reviewers to identify publications providing evidence on the quality of reporting of survey research in the health science literature. We identified the aspects of reporting survey research that were addressed in these evaluative studies and summarized their results descriptively.

### Part 3: Assessment of Quality of Survey Reporting

The results from Part 1 and Part 2 identified items critical to reporting survey research and were used to inform the development of a data abstraction tool. Thirty-two items were deemed most critical to the reporting of survey research on that basis. These were compiled and categorized into a draft data abstraction tool that was reviewed and modified by all the authors, who have expertise in research methodology and survey research. The resulting draft data abstraction instrument was piloted by two researchers (CB, SK) on a convenience sample of survey articles identified by the authors. Items were added and removed and the wording was refined and edited through discussion and consensus among the coauthors. The revised final data abstraction tool (Table S1) comprised 33 items.

Aiming for a minimum sample size of 100 studies, we searched the top 15 journals (by impact factor) from each of four broad areas of health research: health science, public health, general/internal medicine, and medical informatics. These categories, identified through Web of Science, were known to publish survey research and covered a broad range of the biomedical literature. An Ovid MEDLINE search of these 57 journals (three were included in more than one topic area) included Medical Subject Heading (MeSH) terms (“Questionnaires,” “Data Collection,” and “Health Surveys”) and keyword terms (“survey” and “questionnaire”). The search was limited to studies published between January 2008 and February 2009.

We defined a survey as a research method by which information is gathered by asking people questions on a specific topic and the data collection procedure is standardized and well defined. The information is gathered from a subset of the population of interest with the intent of generating summary statistics that are generalisable to the larger population [1],[2].

Two reviewers (CB, SK) independently screened all citations (title and abstract) to determine whether the study used a survey instrument consistent with our definition. The same reviewers screened all full-text articles of citations meeting our inclusion criteria, and those whose eligibility remained unclear. We included all primary reports of self-administered surveys, excluding secondary analyses, longitudinal studies, or surveys that were administered openly through the web (i.e., studies that lacked a clearly defined sampling frame). Duplicate data extraction was completed by the two reviewers. Inconsistencies were resolved by discussion and consensus.

## Results

### Part 1: Identification of Current Guidance for Survey Research – “Instructions to Authors”

Of the 165 journals searched, 154 (93.3%) did not provide any guidance on survey reporting. Of these 154, 126 (81.8%) have published survey research, while 28 have not. Of the 11 journals providing some guidance, eight provided a brief phrase, statement of guidance, or reference; and three provided more substantive guidance, including more than one directive or statement. Examples are provided in Table 1. Although no reporting guidelines for survey research were identified, several journals referenced the EQUATOR Network's web site. The EQUATOR Network includes two papers relevant to reporting survey research [13],[14].

**Table 1:** Instructions to authors—Examples of relevant text per category.

Category	Examples from Instructions to Authors
**No guidance**	“[*Journal name*] does not publish surveys, papers that focus on patient satisfaction, quality assurance, or didactics.”
	“Regular articles include but are not limited to clinical trials, interventional studies, cohort studies, case-control studies, epidemiologic assessments, and surveys.”
**One statement, directive or reference(s)**	“If appropriate, include how many participants were assessed out of those enrolled, e.g. what was the response rate for a survey.”
	“All randomized controlled trials should include the results of intention-to-treat analysis, and all surveys should include response rates.”
	“The results should include: … the number of patients/hips in the updated series who were examined, the number who responded to questionnaires, and the number with available radiographs…”
**Guidance**	“Survey Research. Manuscripts reporting survey data, such as studies involving patients, clinicians, the public, or others, should report data collected as recently as possible, ideally within the past 2 years [ref]. Survey studies should have sufficient response rates (generally at least 60%) and appropriate characterization of nonresponders to ensure that nonresponse bias does not threaten the validity of the findings. For most surveys, such as those conducted by telephone, personal interviews (e.g., drawn from a sample of households), mail, e-mail, or via the Web, authors are encouraged to report the survey outcome rates by using standard definitions and metrics, such as those proposed by the American Association for Public Opinion Research [ref]”

The EQUATOR Network also links to the STROBE (STrengthening the Reporting of OBservational studies in Epidemiology) Statement (www.strobe-statement.org). Although the STROBE Statement includes cross-sectional studies, a class of studies that subsumes surveys, not all surveys are epidemiological. Additionally, STROBE does not include Methods' and Results' reporting characteristics that are unique to surveys (Table S1).

### Part 1: Identification of Current Guidance for Survey Research - Published Survey Reporting Guidelines

**Figure 1:**
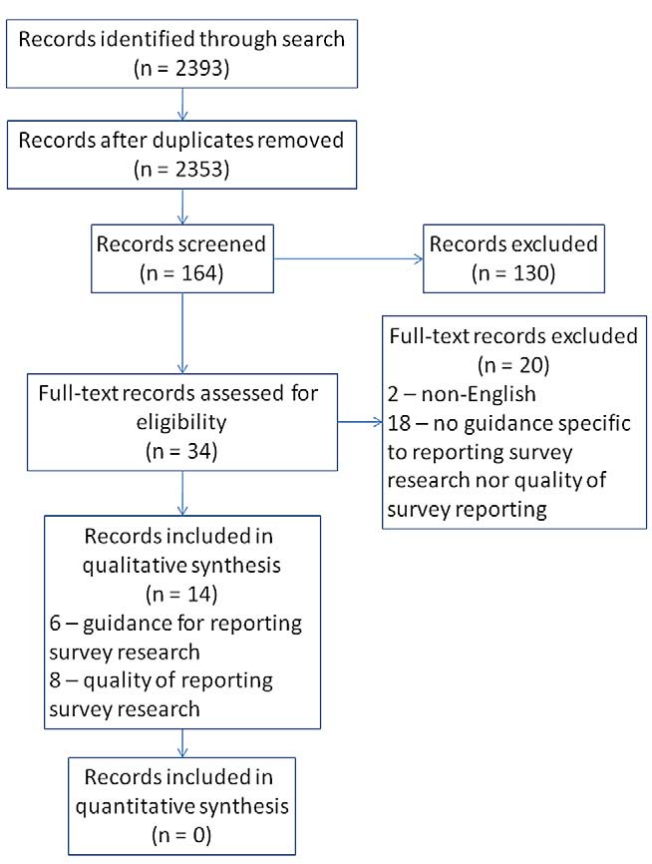
Flow diagram of records and reports—Guidelines for survey research and evidence on the quality of reporting of surveys.

Our search identified 2,353 unique records (Figure 1), which were title-screened. One-hundred sixty-four records were included in the abstract screen, from which 130 were excluded. The remaining 34 records were retrieved for full-text screening to determine eligibility. There was substantial agreement between reviewers across all the screening phases (kappa  =  0.73; 95% CI 0.69–0.77).

We identified five papers [13]–[17] and one internet site [18] that provided guidance on the reporting of survey research. None of these sources reported using valid measures or explicit methods for development. In all cases, in addition to more descriptive details, the guidance was presented in the form of a numbered or bulleted checklist. One checklist was excluded from our descriptive analysis as it was very specific to the reporting of internet surveys [16]. Two checklists were combined for analysis because one [14] was a slightly modified version of the other [17].

Amongst the four checklists, 38 distinct reporting items were identified and grouped in eight broad themes: background, methods, sample selection, research tool, results, response rates, interpretation and discussion, and ethics and disclosure (Table 2). Only two items appeared in all four checklists: providing a description of the questionnaire instrument and describing the representativeness of the sample to the population of interest. Nine items appear in three checklists, 17 items appear in two checklists, and 10 items appear in only one checklist.

**Table 2:** Checklist items for reporting survey research.

Reporting Item	Kelley [13]	Burns [14]	Draugalis [15]	AAPOR [18]
**Background**				
Justification of research method	x	x	x	
Background literature review	x	x		
Explicit research question	x	x	x	
Clear study objectives		x	x	x
**Methods**				
Description of methods used for data analysis	x		x	x
Method of questionnaire administration		x		x
Location of data collection	x	x		x
Dates of data collection				x
Number and types of contact	x		x	x
Methods sufficiently described for replication		x	x	
Evidence of reliability			x	
Evidence of validity			x	
Methods for verifying data entry			x	
Use of a codebook			x	x
**Sample selection**				
Sample size calculation			x	x
Representativeness	x	x	x	x
Method of sample selection	x		x	x
Description of population and sample frame				x
**Research tool**				
Description of the research tool	x	x	x	x
Description - development of research tool	x		x	
Instrument pretesting	x		x	
Instrument reliability and validity	x	x	x	
Scoring methods			x	x
**Results**				
Results of research presented	x	x		
Results address objectives	x	x		
Clear description - results based on part sample				x
Generalisability			x	x
**Response rates**				
Response rate stated	x		x	x
How response rate was calculated			x	x
Discussion of nonresponse bias			x	
All respondents accounted for		x		x
**Interpretation and discussion**				
Interpret and discuss findings	x	x		
Conclusions and recommendations	x	x		
Limitations	x	x		
**Ethics and disclosure**				
Consent	x		x	
Sponsorship				x
Research ethics approval			x	
Evidence of ethical treatment of human subjects			x	

### Part 2: Systematic Review of Published Studies on the Quality of Survey Reporting

Screening results are presented in Figure 1. Eight papers were identified that addressed the quality of reporting of some aspect of survey research. Five studies [19]–[23] addressed the reporting of response rates; three evaluated the reporting of non-response analyses in survey research [20],[21],[24]; and two assessed the degree to which authors make their survey instrument available to readers (Table 3) [25],[26].

**Table 3:** Systematic review – evidence on the of quality of reporting of survey research.

Reporting Criteria	Reference	Journals Reviewed	Number of Surveys	Results
Response rates	Badger 2005 [19]	3 nursing journals 2002	270	49% did not report a response rate or provide sufficient sample disposition to calculate
	Smith 2002 [23]	8 journals: political science, sociology, survey research 1998–2001	571	60% did not provide a response rate; lower for survey research (54%) than sociology (59%) or political science (73%)
	Asch 1997 [20]	111 Medical Journals 1991	321 (178 articles)	30% did not report a response rate or provide sufficient sample disposition to calculate
	Johnson 2003 [22]	9 social science and 9 health science journals 2000–2003	95	5% did not report a response rate. Quality of response rate varied by mode of administration - mail surveys providing a more complete sample disposition
	Cummings 2002 [21]	Physician surveys 1986–1995	257	5% did not report a response rate; of those that did, a further 3% did not provide the number of individuals in the sample or the number responding
Non-response analysis	Werner 2007 [24]	9 management journals 2000–2004	705	31% reported non-response analyses
	Asch 1997 [20]	111 Medical Journals 1991	321 (178 articles)	26% reported non-response analyses
	Cummings 2001 [21]	Physician surveys 1986–1995	257	18% reported non-response analyses
Survey instrument	Schilling 2006 [25]	3 general medicine journals 2000–2003	93	8% provided access to the questionnaire. When corresponding authors were contacted, 46% failed to provide the questionnaire despite repeated contact
	Rosen 2006 [26]	4 epidemiological journals 2005	71	85% did not provide access to the complete questionnaire. 13% did not indicate the type of questionnaire (i.e., interviewer or self-administered); and of those indicating the type 10% did not report mode of administration

### Part 3: Assessment of Quality of Survey Reporting from the Biomedical Literature

**Figure 2:**
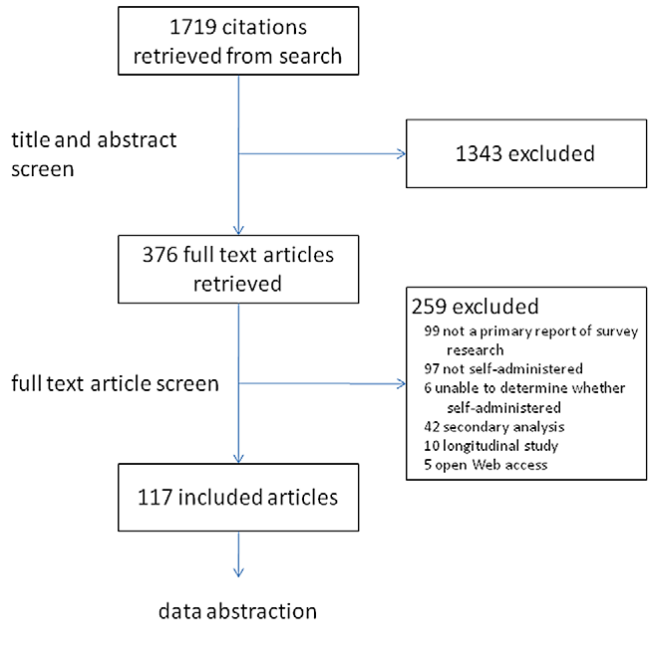
Identification process for article selection—Review of published reports of self-reported surveys.

Our search identified 1,719 citations: 1,343 citations were excluded during title/abstract screening because these studies did not use a survey instrument as their primary research tool. Three hundred seventy-six citations were retrieved for full-text review. Of those, 259 did not meet our eligibility criteria; reasons for their exclusion are reported in Figure 2. The remaining 117 articles, reporting results from self-administered surveys, were retained for data abstraction.

The 117 articles were published in 34 different journals: 12 journals from health science, seven from medical informatics, 10 from general/internal medicine, and eight from public health (Table S2). The median number of pages per study was 8 (range 3–26). Of the 33 items that were assessed using our data abstraction form, the median number of items reported was 18 (range 11–25).

### Reporting Characteristics: Title, Abstract, and Introduction

The majority (113 [97%]) of articles used the term “survey” or “questionnaire” in the title or abstract; four articles did not use a term to indicate that the study was a survey. While all of the articles presented a background to their research, 17 (15%) did not identify a specific purpose, aim, goal, or objective of the study. (Table 4)

**Table 4:** Items reported by 117 included articles.

Criteria	Category	Number (%)
**Title and Abstract**		
Design of study stated	Both title and abstract	90 (77)
	Either title or abstract	23 (20)
	Not stated	4 (3)
**Introduction**		
Background provided	Yes	117 (100)
Purpose/aim of paper explicitly stated	Yes	100 (85)
	No	17 (15)
**Methods**		
***Research Tool***		
Description of the questionnaire	Questionnaire provided	16 (14)
	Core questions provided	25 (21)
	One complete question provided	36 (31)
	Questions not provided	40 (34)
Existing tool, psychometric properties presented	Yes	12 (10)
	No	40 (34)
	Not applicable	65 (56)
Existing tool, references to original work provided	Yes	50 (43)
	No	2 (2)
	Not applicable	65 (56)
New tool, procedures to develop and pre-test provided	Yes	20 (17)
	No	91 (78)
	Not applicable	6 (5)
New tool, reliability and validity reported	Both	3 (3)
	Reliability only	11 (9)
	Validity only	8 (7)
	Neither	88 (75)
	Not applicable	7 (6)
Description of the scoring procedures provided	Yes	38 (32)
	No	63 (54)
	Not applicable	16 (14)
***Sample Selection***		
Description of survey population and sample frame	Both	4 (3)
	Survey population	43 (37)
	Sample frame	63 (54)
	Neither	7 (6)
Description of representativeness of the sample	Yes	13 (11)
	No	104 (89)
Sample size calculation or rationale/justification presented	Yes	7 (6)
	No	110 (94)
***Survey Administration***		
Mode of administration	Mail	67 (57)
	In-person self-administered	13 (11)
	Mixed-mode	14 (12)
	Not explicitly stated	23 (20)
Information on the type and number of contacts provided	Type and number	61 (52)
	Type only	15 (13)
	No information	41 (35)
Information on financial incentives provided	Yes	27 (23)
	No	90 (77)
Description of who approached potential participants	Yes	15 (13)
	No	102 (87)
***Analysis***		
Method of data analysis described	Adequate	50 (43)
	Inadequate	55 (47)
	No description	12 (10)
Method for analysis of nonresponse error provided	Yes	15 (13)
	No	102 (87)
Method for calculating response rate provided	Yes	5 (4)
	No	112 (96)
Definitions for complete versus partial completions provided	Yes	5 (4)
	No	112 (96)
Methods for handling item missing data provided	Yes	13 (11)
	No	104 (89)
**Results**		
Response rate reported	Yes, defined	89 (76)
	Yes, not defined	20 (17)
	Partial information	6 (5)
	No information	2 (2)
All respondents accounted for	Yes	15 (13)
	No	102 (87)
Information on how non-respondents differ from respondents provided	Yes	33 (28)
	Issue addressed	4 (3)
	No information	80 (68)
Results clearly presented	Yes – complete	42 (36)
	Yes – partial	39 (33)
	No	36 (31)
Results address objectives	Yes	114 (97)
	No	3 (3)
**Discussion**		
Results summarized referencing study objectives	Yes	117 (100)
Strengths of the study stated	Yes	27 (23)
	No	90 (77)
Limitations of the study stated	Yes	110 (94)
	No	7 (6)
Generalisability of results discussed	Yes	47 (40)
	No	70 (60)
**Ethical Quality Indicators**		
Study funding reported	Yes	86 (74)
	No	31 (27)
Research Ethics Board (REB) review reported	Yes	69 (59)
	Reported REB exempt	8 (7)
	No	40 (34)
Subject consent procedures reported	Yes	27 (23)
	Reported waiver of informed consent	2 (2)
	No	88 (75)

### Reporting Characteristics: Methods

Approximately one-third (40 [34%]) of survey research reports did not provide access to the questionnaire items used in the study in either the article, appendices, or an online supplement. Of those studies that reported the use of existing survey questionnaires, the majority (40/52 [77%]) did not report the psychometric properties of the tool (although all but two did reference their sources). The majority of studies that developed a novel questionnaire (91/111 [82%]) failed to clearly describe the development process and/or did not describe the methods used to pre-test the tool; the majority (89/111 [80%]) also failed to report the reliability or validity of a newly developed survey instrument. For those papers which used survey instruments that required scoring (n = 95), 63 (66%) did not provide a description of the scoring procedures.

With respect to a description of sample selection methods, 104 (89%) studies did not describe the sample's representativeness of the population of interest. The majority (110 [94%]) of studies also did not present a sample size calculation or other justification of the sample size.

There were 23 (20%) papers for which we could not determine the mode of survey administration (i.e., in-person, mail, internet, or a combination of these). Forty-one (35%) articles did not provide information on either the type (i.e. phone, e-mail, postal mail) or the number of contact attempts. For 102 (87%) papers, there was no description of who was identified as the organization/group soliciting potential research subjects for their participation in the survey.

Twelve (10%) papers failed to provide a description of the methods used to analyse the data (i.e., a description of the variables that were analysed, how they were manipulated, and the statistical methods used). However, for a further 55 (47%) studies, the data analysis would be a challenge to replicate based on the description provided in the research report. Very few studies provided methods for analysis of non-response error, calculating response rates, or handling missing item data (15 [13%], 5 [4%], and 13 [11%] respectively). The majority (112 [96%]) of the articles did not provide a definition or cut-off limit for partial completion of questionnaires.

### Reporting Characteristics: Results

While the majority (89 [76%]) of papers provided a defined response rate, 28 studies (24%) failed to define the reported response rate (i.e., no information was provided on the definition of the rate or how it was calculated), provided only partial information (e.g., response rates were reported for only part of the data, or some information was reported but not a response rate), or provided no quantitative information regarding a response rate. The majority (104 [87%]) of studies did not report the sample disposition (i.e., describing the number of complete and partial returned questionnaires according to the number of potential participants known to be eligible, of unknown eligibility, or known to be ineligible). More than two-thirds (80 [68%]) of the reports provided no information on how non-respondents differed from respondents.

### Reporting Characteristics: Discussion and Ethical Quality Indicators

While all of the articles summarized their results with regard to the objectives, and the majority (110 [94%]) described the limitations of their study, most (90 [77%]) did not outline the strengths of their study and 70 (60%) did not include any discussion of the generalisability of their results.

When considering the ethical quality indicators, reporting was varied. While three-quarters (86 [74%]) of the papers reported their source of funding, approximately the same proportion (88 [75%]) did not include any information on consent procedures for research participants. One-third (40 [34%]) of papers did not report whether the study had received research ethics board review.

## Discussion

Our comprehensive review, to identify relevant guidance for survey research and evidence on the quality of reporting of surveys, substantiates the need for a reporting guideline for survey research. Overall, our results show that few medical journals provide guidance to authors regarding survey research. Furthermore, no validated guidelines for reporting surveys currently exist. Previous reviews of survey reporting quality and our own review of 117 published studies revealed that many criteria are poorly reported.

Surveys are common in health care research; we identified more than 117 primary reports of self-administered surveys in 34 high-impact factor journals over a one-year period. Despite this, the majority of these journals provided no guidance to authors for reporting survey research. This may stem, at least partly, from the fact that validated guidelines for survey research do not exist and that recommended quality criteria vary considerably. The recommended reporting criteria that we identified in the published literature are not mutually exclusive, and there is perhaps more overlap if one takes into account implicit and explicit considerations. Regardless of these limitations, the lack of clear guidance has contributed to inconsistency in the literature; both this work and that of others [19]–[26] shows that key survey quality characteristics are often under-reported.

Self-administered sample surveys are a type of observational study and for that reason they can fall within the scope of STROBE. However, there are methodological features relevant to sample surveys that need to be highlighted in greater detail. For example, surveys that use a probability sampling design do so in order to be able to generalise to a specific target population (many other types of observational research may have a more “infinite” target population); this emphasizes the importance of coverage error and non-response error – topics that have received attention in the survey literature. Thus, in our data abstraction tool, we placed emphasis on specific methodological details excluded from STROBE – such as non-response analysis, details of strategies used to increase response rates (e.g., multiple contacts, mode of contact of potential participants), and details of measurement methods (e.g., making the instrument available so that readers can consider questionnaire formatting, question framing, choice of response categories, etc.).

Consistent with previous work [25],[26], fully one-third of our sample failed to provide access to any survey questions used in the study. This poses challenges both for critical analysis of the studies and for future use of the tools, including replication in new settings. These challenges will be particularly apparent as the articles age and study authors become more difficult to contact [25].

Assessing descriptions of the study population and sampling frame posed particular challenges in this study. It was often unclear whom the authors considered to be the population of interest. To standardise our assessment of this item, we used a clearly delineated definition of “survey population” and “sampling frame” [3],[27]. A survey reporting guideline could help this issue by clearly defining the difference between the terms and descriptions of “population” and “sampling frame.”

Our results regarding reporting of response rates and non-response analysis were similar to previously published studies [19]–[24]. In our sample, 24% of papers assessed did not provide a defined response rate and 68% did not provide results from non-response analysis. The wide variation in how response rates are reported in the literature is perhaps a historical reflection of the limited consensus or explicit journal policy for response rate reporting [22],[28],[29]. However, despite lack of explicit policies regarding acceptable standards for response rates or the reporting of response rates, journal editors are known to have implicit policies for acceptable response rates when considering the publication of surveys [17],[22],[29],[30]. Given the concern regarding declining response rates to surveys [31], there is a need to ensure that aspects of the survey's design and conduct are well reported so that reviewers can adequately assess the degree of bias that may be present and allay concerns over the representativeness of the survey population.

With regard to the ethical quality indicators, sources of study funding were often reported (74%) in this sample of articles. However, the reporting of research ethics board approval and subject consent procedures were reported far less often. In particular, the reporting of informed consent procedures was often absent in studies where physicians, residents, other clinicians or health administrators were the subjects. This finding may suggest that researchers do not perceive doctors and other health-care professionals and administrators to be research subjects in the same way they perceive patients and members of the public to be. It could also reflect a lack of current guidelines that specifically address the ethical use of health services professionals and staff as research subjects.

Our research is not without limitations. With respect to the review of journals' “Instructions to Authors,” the study was cross-sectional in contrast with the dynamic nature of web pages. Since our searches in early 2009, several journals have updated their web pages. It has been noted that at least one has added a brief reference to the reporting of survey research.

A second limitation is that our sample included only the contents of “Instructions to Authors” web pages for higher-impact factor journals. It is possible that journals with lower impact factors contain guidance for reporting survey research. We chose this approach, which replicates previous similar work [12], to provide a defensible sample of journals.

</?twb=.3w?>Third, the problem of identifying non-randomised studies in electronic searches is well known and often related to the inconsistent use of terminology in the original papers. It is possible that our search strategy failed to identify relevant articles. However, it is unlikely that there is an existing guideline for survey research that is in widespread use, given our review of actual surveys, instructions to authors, and reviews of reporting quality.

Fourth, although we restricted our systematic review search strategy to two health science databases, our hand search did identify one checklist that was not specific to the health science literature [18]. The variation in recommended reporting criteria amongst the checklists may, in part, be due to variation in the different domains (i.e., health science research versus public opinion research).

Additionally, we did not critically appraise the quality of evidence for items included in the checklists nor the quality of the studies that addressed the quality of reporting of some aspect of survey research. For our review of current reporting practices for surveys, we were unable to identify validated tools for evaluation of these studies. While we did use a comprehensive and iterative approach to develop our data abstraction tool, we may not have captured information on characteristics deemed important by other researchers. Lastly, our sample was limited to self-administered surveys, and the results may not be generalisable to interviewer-administered surveys.

Recently, Moher and colleagues outlined the importance of a structured approach to the development of reporting guidelines [7]. Given the positive impact that reporting guidelines have had on the quality of reporting of health research [8]–[11], and the potential for a positive upstream effect on the design and conduct of research [32], there is a fundamental need for well-developed reporting guidelines. This paper provides results from the initial steps in a structured approach to the development of a survey reporting guideline and forms the foundation for our further work in this area.

In conclusion, there is limited guidance and no consensus regarding the optimal reporting of survey research. While some key criteria are consistently reported by authors publishing their survey research in peer-reviewed journals, the majority are under-reported. As in other areas of research, poor reporting compromises both transparency and reproducibility, which are fundamental tenets of research. Our findings highlight the need for a well developed reporting guideline for survey research – possibly an extension of the guideline for observational studies in epidemiology (STROBE) – that will provide the structure to ensure more complete reporting and allow clearer review and interpretation of the results from surveys.

## Supporting Information

Table S1 Data abstraction tool items and overlap with STROBE.
(DOC)

Table S2 Journals represented by 117 included articles.
(DOC)

Text S1 Ovid MEDLINE search strategy.
(DOC)
