# Supplementary material for: Reporting Guidelines for Survey Research: An Analysis of Published Guidance and Reporting Practices
Source: PLoS Med. 2011 Aug 2;8(8):e1001069. doi: 10.1371/journal.pmed.1001069 (PMC3149080; doi:10.1371/journal.pmed.1001069)
Supplement: Table S1 — Data abstraction tool items and overlap with STROBE. (DOC) [file pmed.1001069.s001.doc]

Table S1 Data abstraction tool items and overlap with STROBE

| **Category** | **Item** | **Overlap with STROBE** |
| --- | --- | --- |
| **Title and Abstract** | Is the design of the study design stated in the title and/or abstract? | Indicate the study’s design with a commonly used term in the title or the abstract |
|  |  | Provide in the abstract an informative and balanced summary of what was done and what was found |
| **Introduction** | Is there an explanation of why the research is necessary, placing the study in context of previous work in relevant fields? | Explain the scientific background and rationale for the investigation being reported |
|  | Is the purpose or aim of the paper explained? | State specific objectives, including any prespecified hypotheses |
| **Methods** |  | Present key elements of study design early in the paper |
|  |  |  |
| *Research Tool* | Is the questionnaire described? |  |
|  | If an existing tool was used, are its psychometric properties presented? |  |
|  | If an existing tool was used, are references to the original work provided? |  |
|  | If a new tool was used, are the procedures used to develop and pre-test it provided? |  |
|  | If a new tool was used, have its reliability and validity been reported? |  |
|  | Is a description of the scoring procedures provided? |  |
|  |  | Describe the setting, locations, and relevant dates, including periods of recruitment, exposure, follow-up, and data collection |
| *Sample Selection* | Is there a description of the survey population and the sample frame used to identify this population? | Give the eligibility criteria, and the sources and methods of selection of participants |
|  | Do the authors provide a description of how representative the sample is of the underlying population? |  |
|  | Is a sample size calculation or rationale/justification for the sample size presented? | Explain how the study size was arrived at |
| *Survey Administration* | Mode of administration? |  |
|  | Do the authors provide information on the type of contact and how many attempts were made to contact subjects (i.e., prenotification by letter or telephone, reminder postcard, duplicate questionnaire with reminder etc.)? |  |
|  | Do the authors report whether incentives were provided (financial or other)? |  |
|  | Is there a description of who approached potential participants (e.g., identification of who signed the covering letter)? |  |
|  |  | Clearly define all outcomes, exposures, predictors, potential confounders, and effect modifiers. Give diagnostic criteria, if applicable |
|  |  | For each variable of interest, give sources of data and details of methods of assessment (measurement). Describe comparability of assessment methods if there is more than one group |
|  |  | Describe any efforts to address potential sources of bias |
|  |  | Explain how quantitative variables were handled in the analyses. If applicable, describe which groupings were chosen and why |
| *Analysis* | Is the method of data analysis described? | Describe all statistical methods, including those used to control for confounding |
|  |  | Describe any methods used to examine subgroups and interactions |
|  | Do the authors provide methods for analysis of nonresponse error? |  |
|  | Is the method for calculating response rate provided? |  |
|  | Are definitions provided for complete versus partial completions? |  |
|  | Are the methods for handling item missing data provided? | Explain how missing data were addressed |
|  |  | If applicable, describe analytical methods taking account of sampling strategy |
|  |  | Describe any sensitivity analyses |
| **Results** | Is the response rate reported? |  |
|  | Are all respondents accounted for? | Report numbers of individuals at each stage of study—eg numbers potentially eligible, examined for eligibility, confirmed eligible, included in the study, completing follow-up, and analysed |
|  |  | Give reasons for non-participation at each stage |
|  |  | Consider use of a flow diagram |
|  |  | Give characteristics of study participants (eg demographic, clinical, social) and information on exposures and potential confounders |
|  |  | Indicate number of participants with missing data for each variable of interest |
|  | Is information given on how non-respondents differ from respondents? |  |
|  |  | Report numbers of outcome events or summary measures |
|  |  | Give unadjusted estimates and, if applicable, confounder-adjusted estimates and their precision (eg, 95% confidence interval). Make clear which confounders were adjusted for and why they were included |
|  |  | Report category boundaries when continuous variables were categorized |
|  |  | If relevant, consider translating estimates of relative risk into absolute risk for a meaningful time period |
|  |  | Report other analyses done—eg analyses of subgroups and interactions, and sensitivity analyses |
|  | Are the results clearly presented? |  |
|  | Do the results address the objective(s)? |  |
| **Discussion** | Are the results summarized with reference to the study objectives? | Summarise key results with reference to study objectives |
|  | Are the strengths of the study stated? |  |
|  | Are the limitations of the study (taking into account potential sources of bias or imprecision) stated? | Discuss limitations of the study, taking into account sources of potential bias or imprecision. Discuss both direction and magnitude of any potential bias |
|  |  | Give a cautious overall interpretation of results considering objectives, limitations, multiplicity of analyses, results from similar studies, and other relevant evidence |
|  | Is there explicit discussion of the generalisability (external validity) of the results? | Discuss the generalisability (external validity) of the study results |
| **Ethical Quality Indicators** | Study funding reported? | Give the source of funding and the role of the funders for the present study and, if applicable, for the original study on which the present article is based |
|  | Research Ethics Board (REB) review reported? |  |
|  | Reporting of subject consent procedures? |  |
