# Supplementary material for: Reporting Guidelines for Survey Research: An Analysis of Published Guidance and Reporting Practices
Source: PLoS Med. 2011 Aug 2;8(8):e1001069. doi: 10.1371/journal.pmed.1001069 (PMC3149080; doi:10.1371/journal.pmed.1001069)
Supplement: Table S2 — Journals represented by 117 included articles. (DOC) [file pmed.1001069.s002.doc]

Table S2. Journals represented by 117 included articles

| Journal |
| --- |
|  |
| Academic Medicine |
| American Journal of Epidemiology |
| American Journal of Preventative Medicine |
| American Journal of Public Health |
| Annals of Family Medicine |
| Archives of Internal Medicine |
| BMJ |
| Bulletin of the World Health Organization |
| Cancer Causes Control |
| Cancer Epidemiology Biomarkers & Prevention |
| CMAJ Canadian Medical Association Journal |
| Computers, Informatics, Nursing |
| Drug Safety |
| Evaluation of Health Professionals |
| Health Affairs |
| Health Services Research |
| International Journal of Medical Informatics |
| International Journal of Technology Assessment in Health Care |
| JAMA |
| Journal of Evaluation in Clinical Practice |
| Journal of Pain and Symptom Management |
| Journal of the American Medical Informatics Association |
| Medical Care |
| Medical Care Research and Review |
| Medical Decision Making |
| Medical Education |
| Medicine |
| Methods of Information in Medicine |
| New England Journal of Medicine |
| PLoS Medicine |
| Quality of Life Research |
| The American Journal of Managed Care |
| The American Journal of Medicine |
| Value in Health |
