# Supplementary material for: Reporting Guidelines for Survey Research: An Analysis of Published Guidance and Reporting Practices
Source: PLoS Med. 2011 Aug 2;8(8):e1001069. doi: 10.1371/journal.pmed.1001069 (PMC3149080; doi:10.1371/journal.pmed.1001069)
Supplement: Text S1 — Ovid MEDLINE search strategy. (DOC) [file pmed.1001069.s003.doc]

Text S1 Ovid MEDLINE search strategy

1 data collection/

2 Health Surveys/

3 Questionnaires/

4 (survey$ or questionnaire$).ti.

5 health care surveys/

6 or/1-5

7 Research/

8 Publishing/

9 reporting.tw.

10 (publish$ or publication or research).ti.

11 or/7-10

12 6 and 11

13 reproducibility of results/

14 Quality Control/

15 ((valid$ or reliab$ or quality or accura$) adj2 (result$ or report$ or data)).tw.

16 good practice$.tw.

17 or/13-16

18 12 and 17

19 remove duplicates from 18
